# Renal Tubular Acidosis in the Postpartum Period: A Case Report and Literature Review

**DOI:** 10.1155/2021/6693013

**Published:** 2021-09-07

**Authors:** Carlos E. Duran, Mayra Estacio, Fredy Lozano, Esteban Echeverri, Maria Juliana Riascos, Juan Guillermo Posada, Johanna Schweineberg

**Affiliations:** ^1^Department of Nephrology, Fundacion Valle del Lili, Cali, Colombia; ^2^Health Sciences Faculty, Universidad CES, Cali, Colombia; ^3^Health Sciences Faculty, Universidad del Valle, Cali, Colombia; ^4^Health Sciences Faculty, Universidad ICESI, Cali, Colombia

## Abstract

*Case Presentation*. Distal renal tubular acidosis (dRTA) is characterized by impaired hydrogen ion secretion in the distal nephron resulting either from decreased net activity of the proton pump or from increased luminal membrane hydrogen ion permeability. Typical complications of dRTA include severe hypokalemia, normal anion gap metabolic acidosis, nephrolithiasis, and nephrocalcinosis. The patient is a 25-year-old woman in immediate puerperium with hypokalemia leading to paralysis, and the laboratory findings in this patients were concerning for dRTA. It is rare to encounter this entity during pregnancy, and the impact of this pathology is unknown.

## 1. Case Presentation

A 25-year-old woman in immediate puerperium was admitted to the hospital with proximal weakness that has been increasing progressively until difficulty in mobility and subsequent prostration that occurs seven hours after cesarean.

There were no personal or family history diseases, current medications include ferrous sulfate, and she denied alcohol or recreational drugs. She had one previous pregnancy without complication. This pregnancy was normal, and a cesarean section was performed at 37 weeks for pelvic dystocia.

On physical examination, she was alert and oriented, and her vital signs were normal in the neurological examination with findings of proximal weakness, without alteration in the tendon reflexes and findings of alteration in long tracts or focal neurologic deficit.

Initial laboratory showed normal blood cell counts, severe hypokalemia (potassium, 1.91 mEq/L), and pH of 7.24, bicarbonate at 7.7 mmol/L with normal plasma anion gap (anion GAP: 12, 5 mEq/L) Table 1.

Urine studies showed urinalysis was normal with a pH of 6.5, without urine active sediment. Urinary K: Cr 83.7 mEq/g, urinary anion gap: 24 mmol/L, and urinary osmolar gap is positive Table 2.

Serologic testing was negative for autoimmune causes.

A renal ultrasound revealed normal-sized kidneys with marked increase in the echogenicity in the region of the renal pyramids bilaterally and symmetric consistent with nephrocalcinosis.

Treatment: She received potassium chloride initial by the peripheral vein, and 6 hours after admission, his serum potassium level increased to 2.1 mEq/L with improvement of weakness.

## 2. Discussion

The findings in this patients were concerning for dRTA complicated with severe symptomatic hypokalemia. Distal RTA, also known as type 1 RTA, is characterized by impaired hydrogen ion secretion in the distal nephron for a decreased net activity of the proton pump or increased hydrogen ion permeability of the luminal membrane [1].

The distal renal tubular acidosis should be considered in any patient with an otherwise unexplained normal anion gap metabolic acidosis and hypokalemia. The potassium wasting is generated by the reduction in distal hydrogen ion secretion via several mechanisms such as increase in aldosterone levels due to sodium wasting, as well as metabolic acidosis [1, 2].

This pathology can be associated with nephrocalcinosis, urinary calculi, citraturia, calciuria, osteopenia, osteomalacia, and secondary hyperparathyroidism. It can be seen in the context of immunologic diseases such as Sjögren, systemic lupus erythematosus and rheumatoid arthritis, medications (ifosfamide, amphotericin B, lithium carbonate, and ibuprofen), hypercalciuric conditions (hyperparathyroidism, vitamin D intoxication, and sarcoidosis), and others, including familial causes (medullary sponge kidney and Wilson's disease) [1, 2].

During pregnancy, there are alterations in the way the kidney manages solutes and filtration of solutes looking for an optimal environment for fetal development. The glomerular filtration rate (GFR) increases about 40–50% by week 9 [3]. There is an increase in the urea excretion and, in some cases, variable glycosuria due to a diminished glucose-absorption capacity in the distal tubule. Additionally, low-grade-level proteinuria that generally will not exceed 300 mg/24h is observed [3]. There is also a change in the osmostat, which leads to a decrease in the normal osmolality to 270 mOsm/kg, at which level the secretion of antidiuretic hormone would start, with a concomitant reduction of sodium level of around 4 to 5 mEq/L in normal pregnancy.

Pregnancy is a vasodilated state with an important grade of water retention which also contributes to the plasma sodium reduction [3]. Additionally, in most pregnancies, the rate of potassium excretion is constant with changes in tubular reabsorption that adapt to the GFR [3]. This is accompanied by a physiological compensated respiratory alkalosis due to hyperventilation which is asymptomatic, but poses a disadvantage if metabolic acidosis ensues [4].

The presence of hypokalemia in pregnancy is unusual. There are reported cases of severe hypokalemia in pregnancy associated with renal tubular acidosis in patients with a diagnosis before the pregnancy [5–7]. The impact of dRTA for the mother and child is unknown. The physiological changes in pregnancy could worsen dRTA, and it is believed that due to the increase in GFR during pregnancy, this can lead to an increase of electrolyte loss in urine, including potassium. Women without tubular alterations should be capable of dealing with the increased demand for potassium absorption in the distal tubule. Yet, if symptomatic or asymptomatic alterations exist, the state of increased renal and metabolic demand can overcome the renal potassium absorption renal capacity and lead to an increased kaliuresis and concomitant symptomatic hypokalemia [8]. Additionally, the potassium loss can be understood as a compensatory mechanism for the metabolic acidosis generated by the renal distal tubular acidosis [8].

All cases of hypokalemia in pregnant patients and during the puerperium should be investigated making emphasis in the acid-base balance, the renin/aldosterone axis, and in identifying renal or extrarenal potassium losses. Additionally, the use of certain medications such as steroids, diuretics, insulins, and beta-agonists should be discarded [9]. There are also reports of renal proximal tubular acidosis during pregnancy associated with toluene intoxication due to inhalants abuse, so drug use must always be questioned [5].

Distal tubular acidosis in pregnancy is rare. In some cases, the acid-base and potassium status during follow-up will normalize [5], so it is important to obtain serial potassium and blood gas measurements to determine their behavior in this patient. Generally, these patients are treated with bicarbonate supplementation, potassium citrate, and education for an increased awareness of the delicate and unstable balance of acid-base and potassium homeostasis in people living with renal tubular acidosis.

## Figures and Tables

**Table 1:** Laboratory findings on admission.

Parameter	Result	Reference range
Sodium, mmol/L	146.3	136–145
Potassium, mmol/L	1.9	2.5–5.1
Chloride, mmol/L	128.6	98–107
BUN, mg/dL	8.9	6–20
Creatinine, mg/dL	1.2	0.5–0.95
Calcium ionized, mmol/L	1.43	1.15–1.33
Phosphorus, mg/dL	2.44	2.5–4.5
Magnesium, mg/dL	1.78	1.59–2.56
Arterial blood gas		
pH	7.249	7.35–7.45
PCO2, mm Hg	17.9	35–45
PO2, mm Hg	189.1	83–108
Bicarbonate, mEq/L	7.7	21–28
Osmalality, mOsm/kgH20	313	275–300

**Table 2:** Urine studies.

Parameter	Result
Spot urine chemistry	
Osmolality, mOsm/kg H20	325
Sodium, mmol/L	113.5
Potassium, mmol/L	17.59
Chloride, mmol/L	106.3
Creatinine, mg/dL	21.13
Urea nitrogen, mg/dL	169.30
24-hour urine chemistry	
Volume, mL/24 h	4244
Sodium, mEq/24 h	447.74
Potassium, mEq/24 h	80.51
Chloride, mEq/24 h	451.14
Calcium, mg/24 h	273.74
Magnesium, mg/24 h	87.4

## Data Availability

The data used to support the findings of this study are included within the article.
